# US Cigarette Smoking Disparities by Race and Ethnicity — Keep Going and Going!

**DOI:** 10.5888/pcd20.220375

**Published:** 2023-06-01

**Authors:** René A. Arrazola, Todd Griffin, Natasha Buchanan Lunsford, Deirdre Kittner, Philip Bammeke, Elizabeth A. Courtney-Long, Brian S. Armour

**Affiliations:** 1 Office on Smoking and Health, National Center for Chronic Disease Prevention and Health Promotion, Centers for Disease Control and Prevention, Atlanta, Georgia; 2 Katmai Government Services, Anchorage, Alaska

## Abstract

**Introduction:**

Although current cigarette smoking among US adults decreased from 42.4% in 1965 to 12.5% in 2020, prevalence is higher among certain racial and ethnic groups, including non-Hispanic American Indian and Alaska Native (AIAN) adults.

**Methods:**

We examined trends in current cigarette smoking prevalence, population estimates, and relative disparity among US adults (aged ≥18 y) between 2011 and 2020 by using data from the National Health Interview Survey. SAS-callable SUDAAN was used to obtain prevalence and population estimates, and relative disparity was calculated on the basis of findings in the literature. Trends were significant at *P* < .05.

**Results:**

From 2011 to 2020, linear decreases in prevalence and population estimates were observed for non-Hispanic White (20.6% to 13.3%; 32.1 million to 20.7 million), non-Hispanic Black (19.4% to 14.4%; 5.1 million to 4.0 million), and Hispanic (12.9% to 8.0%; 4.2 million to 3.3 million) adults. For non-Hispanic AIAN adults, prevalence remained around 27%, and a linear increase in the population estimate was observed from 400,000 to 510,000. Relative disparity did not change across racial and ethnic categories.

**Conclusion:**

Linear decreases have occurred between 2011 and 2020 for non-Hispanic White, non-Hispanic Black, and Hispanic adults who smoke, but the number of non-Hispanic AIAN adults who currently smoke has increased by 110,000, and relative disparities persist. To reduce racial and ethnic disparities in smoking, understanding how factors at multiple socioecologic levels impact smoking and helping to inform paths to equitable reach and implementation of tobacco control interventions for all population groups are needed.

**Summary**

**What is already known on this topic?**

Current cigarette smoking prevalence among US adults has decreased from 42.4% in 1965 to 12.5% in 2020, which can be viewed as one of public health’s greatest successes. However, the decline in prevalence has been uneven across races and ethnicities.

**What is added by this report?**

This study is the first to look at trends in current cigarette smoking prevalence, estimated number who smoked, and disparity ratio in the US from 2011 to 2020 using National Health Interview Survey data. Findings indicate that between 2011 and 2020, adult prevalence and estimated number who smoked decreased for non-Hispanic White, non-Hispanic Black, and Hispanic adults, while the estimated number who smoked among non-Hispanic American Indian and Alaska Native adults increased by 110,000.

**What are the implications for public health practice?**

To inform efforts to reduce racial and ethnic disparities in cigarette smoking among US adults, continued exploration is needed to further understand how unique and intersecting factors, at multiple socioecologic levels, affect smoking for aggregated and disaggregated population groups.

## Introduction

Current commercial cigarette smoking (hereinafter “smoking”) prevalence among US adults has decreased from 42.4% in 1965 to 12.5% in 2020 (1). Although this decline can be viewed as one of public health’s greatest successes, racial and ethnic disparities in smoking persist (2). Unjust and unfair systems, practices, policies, and conditions have negatively affected population groups based on race and ethnicity, among other intersectional characteristics, and can help to explain root causes for health disparities (3,4). Some examples of these inequitable drivers of smoking-related health disparities include tobacco industry target marketing, advertising, and use of flavors to entice specific population groups; uneven protections from exposure to secondhand smoke; barriers to health care and treatment of tobacco product use and dependence; and pressures of discrimination, poverty, and other social conditions that can exacerbate smoking and its related health problems (5).

Recent findings show that smoking prevalence remains high among certain groups, including non-Hispanic American Indian and Alaska Native (AIAN) adults (1,6), and research has shown that very little, if any, decrease in smoking prevalence has occurred in certain groups (7–9). Furthermore, information assessing disparities in smoking by race and ethnicity over time at a national level is limited (8–10). Availability of state level results is also limited, and these results show that, in general, the prevalence of current cigarette smoking is lower among Hispanic adults compared with non-Hispanic White and non-Hispanic Black adults (11,12). Furthermore, results from state-level studies showed that in most states, significant declines have occurred in current cigarette smoking prevalence among non-Hispanic White, non-Hispanic Black, and Hispanic adults (11–13). We examined linear trends in smoking prevalence and the number of US adults who smoke cigarettes by race and ethnicity and calculated the relative disparity in smoking prevalence in the last decade.

## Methods

We used data from the 2011–2020 National Health Interview Survey (NHIS), which is an annual, nationally representative, household survey of the noninstitutionalized US civilian population. We used the NHIS Sample Adult component of adults aged 18 years or older. Sample sizes and overall response rates between 2011 and 2020 ranged from 21,153 to 36,697 and 48.9% to 66.3%, respectively; data were weighted to account for complex survey design and provide nationally representative estimates. More detailed information about NHIS and data sets used in this analysis is available elsewhere (14). Institutional review for this research was not obtained because data collection for NHIS was approved by the National Center for Health Statistics Research Ethics Review Board, and analysis of deidentified data from NHIS is exempt from federal regulations for the protection of human research participants. 

Respondents who reported having smoked 100 or more cigarettes during their lifetime and reported that they smoked “every day” or “some days” at the time of survey were classified as people who currently smoke cigarettes. Prevalence and population estimates of smoking were calculated for the following racial and ethnic groups: non-Hispanic White, non-Hispanic Black, non-Hispanic AIAN, non-Hispanic Asian, and Hispanic aggregated population groups. Prevalence and population estimates were also calculated for the overall group, which contains all races and ethnicities including those classified as another non-Hispanic race or multiple races.

SAS-callable SUDAAN software version 11.0.3 (RTI International) was used to obtain prevalence and estimated number of adults who smoke (rounded down to the nearest 10,000) and their corresponding standard errors and 95% CIs. Based on the literature, the relative disparity in smoking prevalence was estimated for each racial and ethnic group by dividing their respective smoking prevalence by the smoking prevalence of the referent group (15). Non-Hispanic Asian respondents were selected as the referent group because this group historically has had the lowest smoking prevalence among reported groups and disparities can be measured from the group with the most favorable rate (16), noting that this carries some advantages and disadvantages (17). In an equitable landscape, a measure of 1 would indicate no disparity, and any number less than or more than 1 would indicate a possible disparity. Providing both prevalence, with which one can compute the absolute difference between groups, and relative disparity helps provide a more comprehensive understanding of the extent to which disparities exist (17). JoinPoint Trend Analysis Software version 4.9.0.0 (National Cancer Institute) was used to determine the presence of any linear trends for both prevalence and estimated number of adults who smoke, as well as to determine relative disparity; linear trends were determined significant at *P* < .05. To enhance the power to detect significant differences in the numbers of non-Hispanic AIAN and non-Hispanic Asian adults who smoke, data were pooled and 3-year moving averages were calculated.

## Results

During 2011–2020, overall adult smoking prevalence declined from 19.0% to 12.5%, and the number of adults who currently smoked cigarettes declined by 13.1 million, from 43.8 million to 30.7 million. Significant linear downward trends were found in prevalence for all assessed racial and ethnic groups except non-Hispanic AIAN (Table 1). From 2011 to 2020, smoking prevalence significantly decreased from 9.9% to 8.0% among non-Hispanic Asian adults, from 19.4% to 14.4% among non-Hispanic Black adults, from 12.9% to 8.0% among Hispanic adults, and from 20.6% to 13.3% among non-Hispanic White adults. Among non-Hispanic AIAN adults, prevalence was 31.5% in 2011 and 27.1% in 2020, but the difference was not significant.

**Table 1:** Percent, Estimated Number, and Disparity Ratio of US Adults Who Currently Smoke Cigarettes, by Race and Ethnicity and Overall, National Health Interview Survey 2011–2020^a^

Race and ethnicity		2011	2012	2013	2014	2015	2016	2017	2018	2019	2020	Linear trend *P* value
Hispanic	% (95% CI)	12.9 (11.8–14.1)	12.5 (11.3–13.7)	12.1 (11.0–13.2)	11.2 (10.2–12.2)	10.1 (9.1–11.0)	10.7 (9.2–12.3)	9.9 (8.6–11.1)	9.8 (8.4–11.2)	8.8 (7.8–9.9)	8.0 (6.9–9.2)	Decreasing .001
	Estimated no.^b^ (95% CI)	42.3 (38.3–46.3)	43.4 (39.1–47.8)	43.2 (38.7–47.6)	40.7 (36.5–44.9)	37.9 (33.6–42.2)	41.5 (33.3–49.6)	38.8 (32.4–45.2)	39.6 (33.3–46.0)	35.4 (30.4–40.3)	33.2 (27.7–38.6)	Decreasing <.001
	Relative disparity^c^ (95% CI)	1.3 (1.1–1.6)	1.2 (1.0–1.4)	1.3 (1.0–1.5)	1.2 (1.0–1.4)	1.4 (1.2–1.8)	1.2 (0.9–1.6)	1.4 (1.1–1.8)	1.4 (1.0–1.9)	1.2 (0.9–1.6)	1.0 (0.8–1.3)	Decreasing .64
NH AIAN	% (95% CI)	31.5 (21.4–41.7)	21.8 (15.0–28.6)	26.1 (18.5–33.7)	29.2 (19.6–38.8)	21.9 (16.6–27.1)	31.8 (24.1–39.5)	24.0 (14.4–33.6)	22.6 (12.0–33.3)	20.9 (9.8–31.9)	27.1 (16.9–37.2)	Decreasing .75
	Estimated no. (95% CI)	4.0 (2.8–5.2)	2.4 (1.6–3.3)	3.3 (2.1–4.4)	3.5 (2.0–5.1)	3.2 (2.0–4.4)	4.9 (3.2–6.6)	4.3 (2.5–6.1)	4.6 (2.6–6.6)	3.7 (2.3–5.1)	5.1 (3.0–7.1)	Increasing .09
	Relative disparity (95% CI)	3.2 (2.2–4.6)	2.0 (1.4–2.9)	2.7 (1.9–3.8)	3.1 (2.1–4.5)	3.1 (2.3–4.3)	3.5 (2.6–4.3)	3.4 (2.1–5.2)	3.2 (1.9–5.5)	2.9 (1.6–5.1)	3.4 (2.2–5.2)	Increasing .11
NH Asian	% (95% CI)	9.9 (8.4–11.4)	10.7 (9.1–12.3)	9.6 (7.9–11.4)	9.5 (7.7–11.2)	7.0 (5.6–8.5)	9.0 (7.1–10.9)	7.1 (5.5–8.8)	7.1 (5.2–8.9)	7.2 (5.4–9.0)	8.0 (6.3–9.8)	Decreasing .006
	Estimated no. (95% CI)	10.8 (9.1–12.5)	12.8 (10.7–15.0)	12.2 (9.8–14.7)	12.4 (9.8–15.0)	9.6 (7.5–11.7)	12.6 (9.6–15.7)	10.4 (7.7–13.1)	10.7 (7.8–13.6)	10.3 (7.6–13.0)	11.8 (8.9–14.6)	Increasing .07
	Relative disparity (95% CI)	1.0 [Reference]										—
NH Black	% (95% CI)	19.4 (18.1–20.8)	18.1 (16.7–19.4)	18.3 (16.8–19.7)	17.5 (16.1–18.8)	16.7 (15.2–18.2)	16.5 (14.7–18.3)	14.9 (13.1–16.6)	14.6 (12.8–16.3)	14.9 (13.3–16.4)	14.4 (12.6 –16.2)	Decreasing <.001
	Estimated no. (95% CI)	51.4 (47.0–55.7)	48.2 (44.1–52.2)	49.9 (45.0–54.7)	48.5 (44.1–52.8)	47.2 (42.6–51.8)	47.3 (41.3–53.3)	43.1 (37.3–48.9)	42.2 (36.4–48.1)	42.0 (36.5–47.4)	40.9 (34.6–47.1)	Decreasing <.001
	Relative disparity (95% CI)	2.0 (1.7–2.3)	1.7 (1.4–2.0)	1.9 (1.6–2.3)	1.8 (1.5–2.3)	2.4 (1.9–3.0)	1.8 (1.5–2.3)	2.1 (1.6–2.7)	2.1 (1.5–2.8)	2.1 (1.6–2.7)	1.8 (1.4–2.3)	Increasing .46
NH White	% (95% CI)	20.6 (19.8–21.4)	19.7 (18.9–20.4)	19.4 (18.6–20.3)	18.2 (17.3–19.1)	16.6 (15.8–17.3)	16.6 (15.9–17.4)	15.2 (14.4–15.9)	15.0 (14.3–15.7)	15.5 (14.9–16.2)	13.3 (12.7–14.0)	Decreasing <.001
	Estimated no. (95% CI)	321.1 (306.0–336.2)	305.0 (290.6–319.4)	303.4 (288.8–317.9)	285.1 (268.1–302.1)	260.1 (246.0–274.1)	261.1 (246.5–275.7)	237.9 (223.9–251.9)	234.7 (221.8–247.5)	240.3 (227.3–253.2)	207.5 (194.9–220.0)	Decreasing <.001
	Relative disparity (95% CI)	2.1 (1.8–2.4)	1.8 (1.6–2.2)	2.0 (1.7–2.4)	1.9 (1.6–2.3)	2.4 (1.9–2.9)	1.9 (1.5–2.3)	2.1 (1.7–2.7)	2.1 (1.6–2.7)	2.2 (1.7–2.8)	1.7 (1.3–2.1)	Decreasing .77
Overall	% (95% CI)	19.0 (18.4–19.6)	18.1 (17.5–18.7)	17.8 (17.2–18.4)	16.8 (16.1–17.4)	15.1 (14.5–15.7)	15.5 (14.8–16.1)	14.0 (13.6–14.6)	13.7 (13.1–14.3)	14.0 (13.4–14.5)	12.5 (11.9–13.0)	Decreasing <.001
	Estimated no. (95% CI)	438.2 (421.2–455.2)	421.0 (405.0–436.9)	421.4 (405.0–437.8)	399.6 (380.8–418.4)	365.0 (349.1–380.8)	377.8 (357.4–398.1)	342.8 (326.1–359.6)	341.5 (325.7–357.4)	340.6 (325.3–355.9)	307.8 (291.2–324.8)	Decreasing <.001

Abbreviations: AIAN, American Indian or Alaska Native; NH, non-Hispanic.

a Overall results contain all responses for all races and ethnicities including those classified as another non-Hispanic race or multiple races.

b Estimated numbers in 100,000, have been rounded down to the nearest 10,000.

c Relative disparity is the ratio of current cigarette smoking prevalence for a specific race and ethnicity to the referent race and ethnicity. A measure of 1 would indicate no disparity, and any number less than or more than 1 would indicate a possible disparity.

From 2011 to 2020, significant linear downward trends were found for 3 of the 5 population groups in estimated number of adults who currently smoke (Table 1). Among non-Hispanic Black adults, the estimated number who smoked decreased from 5.1 million in 2011 to 4.0 million in 2020. The estimated number of Hispanic and non-Hispanic White adults who smoke decreased from 4.2 million to 3.3 million and from 32.1 million to 20.7 million, respectively. The non-Hispanic Asian and non-Hispanic AIAN estimated number of adults who smoke increased from 1.0 million to 1.1 million and from 0.4 million to 0.5 million, respectively; however, neither of these changes was significant.

Despite decreases in prevalence of smoking among 4 of 5 racial and ethnic categories (Table 1), compared with non-Hispanic Asian adults, the relative disparity for the remaining groups remained unchanged over the study period. The relative disparity for non-Hispanic White adults was 2.1 in 2011 and 1.7 in 2020. For non-Hispanic Black adults, the relative disparity was 2.0 in 2011 and 1.8 in 2020. For Hispanic adults, the relative disparity was 1.3 in 2011 and 1.0 in 2020, and for non-Hispanic AIAN adults the relative disparity was 3.2 in 2011 and 3.4 in 2020.

Pooled data indicated a significant increase in the numbers of non-Hispanic AIAN adults who smoke (3-year moving average, 120,000) and significant decreases in the prevalence (3-year moving average, from 10.1% to 7.4%) and numbers (3-year moving average, 100,000) of non-Hispanic Asian adults who smoke (Table 2). Results from the 3-year moving average also indicated that relative disparity significantly increased among non-Hispanic AIAN and non-Hispanic Black adults who smoke.

**Table 2:** 3-Year Moving Average for Percent, Estimated Number, and Disparity Ratio of US Adults Who Currently Smoke Cigarettes by Race and Ethnicity and Overall, National Health Interview Survey, 2011–2020^a^

Race and ethnicity		2011–2013	2012–2014	2013–2015	2014–2016	2015–2017	2016–2018	2017–2019	2018–2020	Linear trend *P* value
Hispanic	% (95% CI)	12.5 (11.9–13.2)	11.9 (11.3–12.6)	11.1 (10.5–11.7)	10.6 (10.0–11.4)	10.2 (9.5–11.0)	10.1 (9.3–11.0)	9.5 (8.8–10.2)	8.9 (8.2–9.6)	Decreasing <.001
	Estimated no.^b ^(95% CI)	42.9 (40.5–45.4)	42.4 (39.9–44.9)	40.5 (38.1–43.0)	40.0 (36.6–43.3)	39.3 (35.6–43.1)	39.9 (35.9–44.0)	37.9 (34.4–41.3)	36.0 (32.8–39.3)	Decreasing <.001
	Relative disparity^c ^(95% CI)	1.2 (1.1–1.4)	1.2 (1.1–1.3)	1.3 (1.1–1.4)	1.3 (1.1–1.4)	1.3 (1.1–1.5)	1.3 (1.1–1.5)	1.3 (1.1–1.6)	1.2 (1.0–1.4)	Increasing .35
NH AIAN	% (95% CI)	26.7 (22.2–31.7)	25.8 (21.3–30.8)	25.5 (21.4–30.0)	27.6 (23.5–32.2)	25.9 (21.4–30.9)	25.7 (20.3–32.1)	22.5 (17.0–29.2)	23.5 (17.9–30.4)	Decreasing .13
	Estimated no. (95% CI)	3.2 (2.6–3.8)	3.0 (2.3–3.7)	3.3 (2.5–4.0)	3.8 (3.0–4.7)	4.1 (3.2–5.0)	4.5 (3.5–5.6)	4.1 (3.1–5.2)	4.4 (3.4–5.5)	Increasing .001
	Relative disparity (95% CI)	2.6 (2.2–3.2)	2.6 (2.1–3.2)	2.9 (2.4–3.6)	3.3 (2.7–4.0)	3.4 (2.7–4.2)	3.3 (2.6–4.4)	3.2 (2.3–4.3)	3.2 (2.4–4.3)	Increasing .02
NH Asian	% (95% CI)	10.1 (9.2–11.1)	9.9 (9.0–10.9)	8.7 (7.8–9.7)	8.5 (7.5–9.5)	7.7 (6.8–8.7)	7.7 (6.7–8.8)	7.1 (6.2–8.2)	7.4 (6.5–8.5)	Decreasing <.001
	Estimated no. (95% CI)	11.9 (10.7–13.1)	12.5 (11.1–13.8)	11.4 (10.0–12.8)	11.5 (10.0–13.0)	10.8 (9.3–12.4)	11.2 (9.5–12.9)	10.4 (8.8–12.0)	10.9 (9.2–12.5)	Decreasing .008
	Relative disparity (95% CI)	1.0 [Reference]								—
NH Black	% (95% CI)	18.6 (17.8–19.4)	17.9 (17.1–18.7)	17.5 (16.7–18.3)	16.9 (16.0–17.8)	16.0 (15.1–17.0)	15.3 (14.3–16.4)	14.8 (13.8–15.8)	14.6 (13.7–15.6)	Decreasing <.001
	Estimated no. (95% CI)	49.8 (47.2–52.3)	48.8 (46.2–51.3)	48.5 (45.8–51.1)	47.6 (44.7–50.5)	45.8 (42.6–49.0)	44.2 (40.8–47.6)	42.4 (39.1–45.7)	41.6 (38.3–45.0)	Decreasing <.001
	Relative disparity (95% CI)	1.8 (1.7–2.0)	1.8 (1.6–2.0)	2.0 (1.8–2.3)	2.0 (1.8–2.3)	2.1 (1.8–2.4)	2.0 (1.7–2.3)	2.1 (1.8–2.4)	2.0 (1.7–2.3)	Increasing .046
NH White	% (95% CI)	19.9 (19.4–20.4)	19.1 (18.6–19.6)	18.1 (17.6–18.6)	17.1 (16.7–17.6)	16.1 (15.7–16.6)	15.6 (15.2–16.0)	15.2 (14.8–15.6)	14.6 (14.2–15.0)	Decreasing <.001
	Estimated no. (95% CI)	309.8 (301.3–318.2)	297.8 (288.9–306.7)	282.8 (274.0–291.6)	268.7 (259.9–277.5)	253.0 (244.7–261.2)	244.5 (236.5–252.5)	237.6 (229.9–245.2)	227.4 (220.0–234.8)	Decreasing <.001
	Relative disparity (95% CI)	2.0 (1.8–2.2)	1.9 (1.7–2.1)	2.1 (1.9–2.3)	2.0 (1.8–2.3)	2.1 (1.8–2.4)	2.0 (1.8–2.3)	2.1 (1.8–2.5)	2.0 (1.7–2.3)	Increasing .27
Overall	% (95% CI)	18.3 (17.9–18.6)	17.5 (17.2–17.9)	16.6 (16.2–16.9)	15.8 (15.4–16.1)	14.8 (14.5–15.2)	14.4 (14.0–14.7)	13.9 (13.6–14.2)	13.4 (13.1–13.7)	Decreasing <.001
	Estimated no. (95% CI)	426.8 (417.3–436.3)	413.9 (404.1–423.8)	395.3 (385.4–405.1)	380.7 (370.1–391.4)	361.8 (351.6–372.1)	354.0 (343.8–364.3)	341.6 (332.4–350.9)	329.9 (320.7–339.1)	Decreasing <.001

Abbreviations: AIAN, American Indian or Alaska Native; NH, non-Hispanic.

a Overall results contain all responses for all races and ethnicities including those classified as another non-Hispanic race or multiple races.

b Estimated numbers in 100,000, have been rounded down to the nearest 10,000.

c Relative disparity is the ratio of current cigarette smoking prevalence for a specific race and ethnicity to the referent race and ethnicity. A measure of 1 would indicate no disparity, and any number less than or more than 1 would indicate a possible disparity.

## Discussion

The decline in smoking prevalence from 2011 to 2020 can, in part, be attributed to the implementation of tobacco control interventions as part of a comprehensive commercial tobacco control program (2,18). Consistent with previous studies (8–12), disparities in smoking prevalence persist. Our results expand on past national results, which found that among racial and ethnic groups there was no significant change in the prevalence of current cigarette smoking among non-Hispanic AIAN adults, and that there were linear decreases in prevalence of current cigarette smoking among non-Hispanic White, non-Hispanic Black, non-Hispanic Asian, and Hispanic adults (9,11). 

Furthermore, our results are similar to previously published national and state-level trend results in which current cigarette smoking tended to be highest among NH-AIAN adults and lowest among NH-Asian and Hispanic adults (9–11) and noting that no differences exist between non-Hispanic White and non-Hispanic Black adults (9,11–13). Our results are consistent with previous publications as illustrated with similar magnitude and direction of current cigarette smoking among adults. This consistency validates our study, which also expands knowledge by including the most recent national data as it relates to cigarette smoking disparities.

Although increased scientific exploration about the drivers of longstanding racial and ethnic disparities are needed, existing research indicates that many multilevel and interacting factors from the socioecologic model contribute to tobacco-related health disparities (19) and that some of the driving factors could be structural and sociocultural differences, in addition to practices related to smoking (20). Another possible reason that might explain smoking disparities is that if tobacco control interventions do not equally and equitably reach and affect all racial and ethnic populations, they have the potential to exacerbate these disparities (18).

Our study has several limitations. First, self-reported responses were not validated by biochemical testing for cotinine; however, a high correlation exists between self-reported smoking and smokeless use and cotinine levels (21). Second, NHIS is limited to the noninstitutionalized US civilian population, limiting generalization to institutionalized populations, imprisoned persons, and persons in the military. Third, caution should be taken in interpreting results of changes between 2019 and 2020 with earlier years of NHIS because changes in weighting and design methods starting with the NHIS 2019 may affect comparisons of weighted survey estimates over time; in 2019, the changes in weighting and design methods affected the measure of overall current cigarette smoking by an increase of 0.5 percentage points when comparing the old weighting methods (13.7%) to the new ones (14.2%) (22). However, NHIS quarterly estimates from first quarter of 2019 through fourth quarter of 2020 do indicate a decline in current cigarette smoking using the new weighting methods (23). Fourth, changes in population size over the period were not accounted for in our estimation. Finally, estimates of adult smoking are based on aggregated racial and ethnic population categories, which may conceal differences in smoking prevalence among population subgroups in these categories (6,20,24).

Overall prevalence and estimated number of adults who smoked have decreased between 2011 and 2020; however, the decline in smoking prevalence is uneven by race and ethnicity. For non-Hispanic White, non-Hispanic Black, and Hispanic adults, there is a decrease in both prevalence and estimated numbers of people who smoke, and for non-Hispanic Asian adults there was a decrease only in prevalence of smoking. For non-Hispanic AIAN adults, the number who currently smoke increased by 110,000. The lack of significant changes in the relative disparity measure indicate that disparities persist and could potentially be increasing among non-Hispanic AIAN and non-Hispanic Black adults who smoke. Additional surveillance can support not just identification of markers of disparity, but also drivers of smoking-related inequities (25). This surveillance includes exploring unique and intersecting factors that drive smoking among intersectional population groups (25). Furthermore, equitable reach and implementation of evidenced-based commercial tobacco control interventions (eg, smoke-free policies, cessation access, tobacco price increases, mass media campaigns) are needed, as is ongoing evidence of their impact for unique and intersectional population groups.
